# Targeting Mycobacterium tuberculosis pH-driven adaptation

**DOI:** 10.1099/mic.0.001458

**Published:** 2024-05-08

**Authors:** Shelby J. Dechow, Robert B. Abramovitch

**Affiliations:** 1 Department of Microbiology, Genetics and Immunology, Michigan State University, East Lansing, MI 48824, USA

**Keywords:** environmental cues, *Mycobacterium tuberculosis*, pathogenesis, therapeutics

## Abstract

*Mycobacterium tuberculosis* (Mtb) senses and adapts to host environmental cues as part of its pathogenesis. One important cue sensed by Mtb is the acidic pH of its host niche – the macrophage. Acidic pH induces widespread transcriptional and metabolic remodelling in Mtb. These adaptations to acidic pH can lead Mtb to slow its growth and promote pathogenesis and antibiotic tolerance. Mutants defective in pH-dependent adaptations exhibit reduced virulence in macrophages and animal infection models, suggesting that chemically targeting these pH-dependent pathways may have therapeutic potential. In this review, we discuss mechanisms by which Mtb regulates its growth and metabolism at acidic pH. Additionally, we consider the therapeutic potential of disrupting pH-driven adaptations in Mtb and review the growing class of compounds that exhibit pH-dependent activity or target pathways important for adaptation to acidic pH.

## *M. tuberculosis* colonization of acidic environments

Bacterial pathogens must adapt to changing environmental conditions to survive inside the host. Pathogens with an intracellular lifestyle are faced with hostile immune responses and must sense and adapt accordingly. *Mycobacterium tuberculosis* (Mtb) colonizes environments that have mildly acidic pH (such as the macrophage phagosome) or are more strongly acidic (such as the phagolysosome or caseum). Following phagocytosis, Mtb initially inhibits fusion of the phagosome and lysosome in resting macrophages, residing in a mildly acidic environment (pH ~6.2) [1]. Arrest of phagosome maturation during Mtb infection can eventually be overcome. Immunological activation of the macrophage results in phagosomal–lysosomal fusion and acidification to ~pH 4.5–5.0 [1,2], whereupon Mtb may restrict its growth in order to survive [1]. The decrease in pH following phagosomal–lysosomal fusion is rapid and occurs within 15–60 min [3]. Mtb can also perforate the phagosome, granting cytosolic access [4,5] which could result in neutralization of the phagosome and allow Mtb to access cytosolic carbon sources that are otherwise absent in the phagosome [6].

Over time, granulomas form around infected macrophages. Inside the granuloma necrosis generates caseum in which extracellular Mtb is found. Caseum is also a site where extracellular Mtb may encounter acidic conditions. Kempker *et al*. measured a median pH of 5.5 in the caseum of pyrazinamide-treated tuberculosis (TB) patients [7]; however, the caseum has been shown to vary in pH across animal models and humans, with most mature granulomas exhibiting a near neutral pH [8,12]. Notably, caseum is rich in cholesterol, triacylglycerol (TAG) and other fatty acids [13], suggesting integration of pH and carbon source utilization may also play a role in caseum. It is also possible that inflammatory damage to lung tissue can limit CO_2_ exhalation, driving localized hypercapnic acidosis [14]. Thus, during infection, Mtb may experience a variety of different environments, with varying pH and nutrients available. As detailed below, the ability of Mtb to respond to environmental pH and available carbon sources has a considerable impact on Mtb replication, survival and drug susceptibility.

## Slowed growth and metabolic remodelling at acidic pH

Mtb is characterized as a slow-growing pathogen and exhibits a wide range of doubling times, from ~20 h *in vitro* to 70 days in mice [15,16]. Our understanding of how Mtb arrests its growth *in vivo* is limited. However, *in vitro* studies of host-relevant stresses (i.e. hypoxia and nutrient starvation) show that Mtb enters a non-replicating persistent (NRP) state, whereupon it completely arrests its growth, remodels its metabolism and becomes more tolerant to antibiotics [17,22]. Parallels with these observations have also been defined in acid stress models *in vitro* [23,27]. Mtb will incrementally slow its growth in rich medium starting at pH 6.4, with complete growth arrest observed at pH 5.0 [27]. Mtb will also completely arrest its growth in minimal media buffered to pH 5.7 in the presence of glycerol as a sole carbon source, a model of NRP referred to as acid growth arrest [25]. Additionally, slowed Mtb growth occurs in mildly acidic (pH 6.0–6.5) defined Sauton medium under elevated Mg^2+^ levels (100 µM), with complete growth arrest observed at low Mg^2+^ levels (10 µM) [26]. Amid extreme acidic culture conditions (pH 4.5), Mtb is able to maintain a relatively neutral intrabacterial pH (~pH 7.2) and maintain viability [24]. This demonstrates that slowed growth is not attributed to intrabacterial acidification and suggests mechanisms are in place by which Mtb regulates growth arrest in response to changes in pH.

Metabolic remodelling is a hallmark of NRP and is observable under *in vitro* environmental stress conditions including acidic pH [25]. The Mtb genome carries an abundance of genes likely to be involved in fatty acid synthesis and degradation [28], which may be important for metabolizing cholesterol and other host lipids as carbon sources during infection [29,32]. Mtb metabolism of cholesterol, TAG and host lipids produces acetyl-CoA, propionyl-CoA, pyruvate and glycerol – metabolic intermediates important for fuelling Mtb carbon metabolism and pathogenesis [32,33]. This suggests environmental stresses, like low pH encountered in the macrophage or caseum, and available host carbon sources may function together to regulate Mtb metabolic pathways. This is supported by *in vitro* studies of acidic pH and host-associated carbon sources which show that acid growth arrest is dependent on the presence of available glycolytic carbon sources (i.e. glucose and glycerol) [25]. Further mechanistic studies of pH-dependent Mtb growth regulation link acidic pH and carbon source availability to a reduced cytoplasm, sulfolipid synthesis and central carbon metabolism remodelling [25]. Interestingly, Mtb grows at acidic pH in the presence of other carbon sources [i.e. phosphoenolpyruvate (PEP), pyruvate, acetate, oxaloacetate (OA) and cholesterol] which function at the intersection of glycolysis and the TCA cycle, known as the anaplerotic node [25]. In addition to cholesterol, other host-relevant lipids that feed into the anaplerotic node – oleic acid and palmitic acid – have been shown to support growth at pH 5.5 [34]. These discoveries suggest that the anaplerotic node is a pH-dependent metabolic switch that may promote Mtb growth on permissive carbon sources at acidic pH. This is further supported by the observation that anaplerosis-associated genes, phosphoenolpyruvate carboxykinase (*pckA*) and isocitrate lyase (*icl*), are induced in an acidic pH-dependent manner [25]. Deletion of *pckA* and *icl* results in reduced Mtb growth at acidic pH [25,35] and substantial bacterial death when grown in the presence of oleic acid at pH 5.0 [34]. Furthermore, carbon source-specific growth arrest at acidic pH appears to be an Mtb-specific adaptation associated with pathogenesis; the non-pathogenic mycobacterium strain, *Mycobacterium smegmatis*, grows equally well at pH 5.7 regardless of carbon source [25]. Together, these data suggest Mtb remodels its metabolism around the anaplerotic node and requires a diverse array of lipid assimilation genes to metabolize the different host-derived carbon sources that feed this node.

## Mtb sensing and gene regulation at acidic pH

While Mtb remodels its carbon metabolism to promote growth at acidic pH [35], it also employs regulatory mechanisms to slow its growth and enter acid growth arrest. *In vitro* and *in vivo* transcriptional profiling studies of Mtb in response to acidic pH show a robust transcriptional response [25,36, 37], supporting that Mtb can sense a low pH environment and modulate its physiology accordingly. Transcriptional studies of the phagosomal acidic pH regulon show significant overlap with the PhoPR two-component regulatory system regulon, which consists of the sensor histidine kinase PhoR and the response regulator PhoP [38]. Specifically, the induction of 25 genes is shared between both regulons [36,38], suggesting some pH-dependent physiologies are controlled by PhoPR. Mutants in *phoP* are attenuated for virulence in infected macrophages, mice and guinea pigs [27,39], further supporting that Mtb regulatory responses to low pH are important for virulence and acid adaptation. Notably, acidic pH and chloride function synergistically to regulate PhoPR [40], and the acidic pH response is orchestrated by several other regulators including PrrA [41], Rv0500A [42], SigE [43], CRP [44], TcrXY [45] and WhiB3 [46,47].

**Fig. 1.**
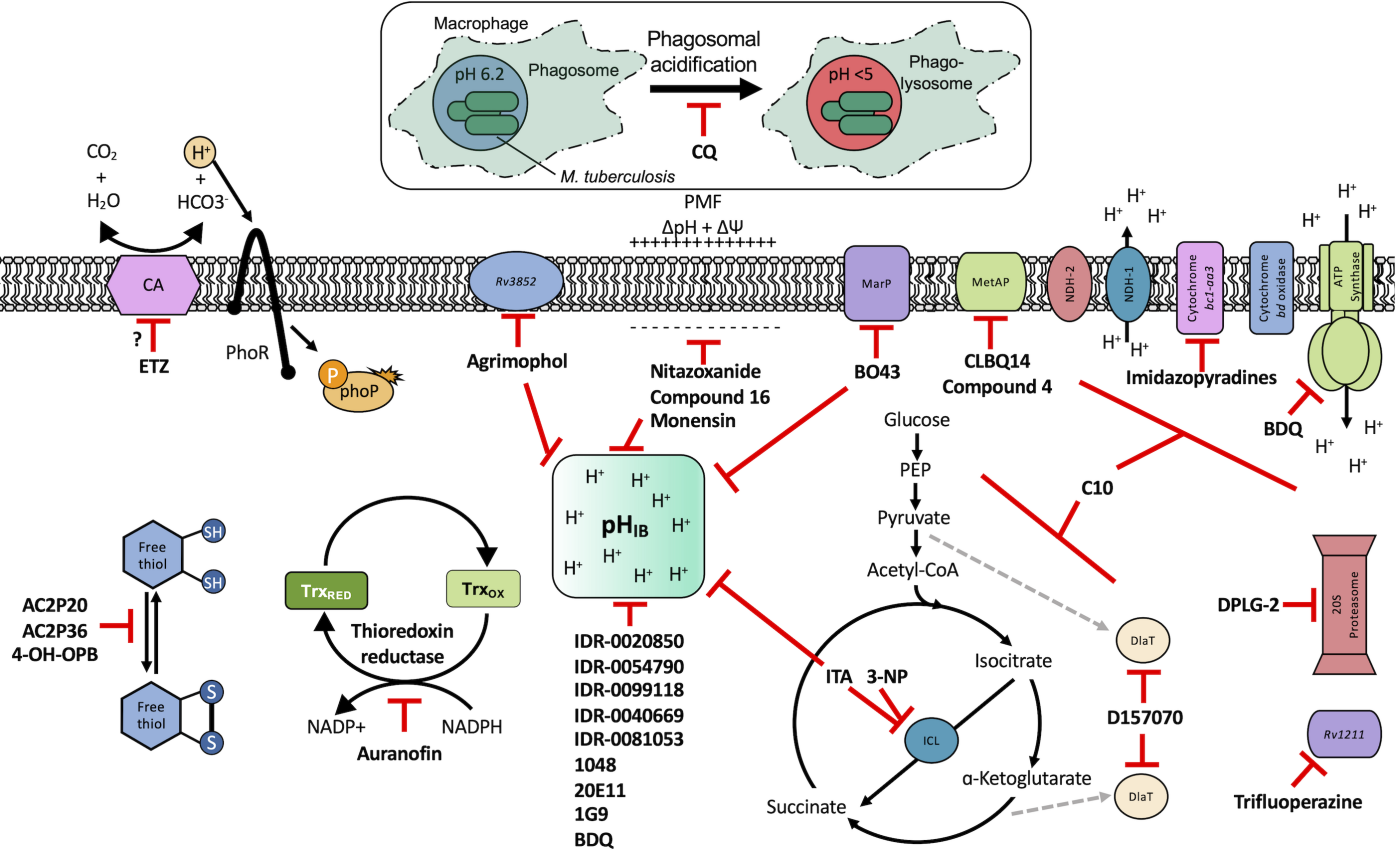
Small molecules targeting *M. tuberculosis* pH-adaptation pathways. Acidic pH modulates key pathways and physiologies involved in redox homeostasis, carbon metabolism and pH homeostasis. This model summarizes known pH-responsive physiological adaptations and small molecules (described in Table 1) that disrupt intrabacterial pH (pH_IB_), membrane potential (ΔΨ), carbon metabolism, redox homeostasis and the electron transport chain (ETC). PhoPR is induced by acidic pH, possibly via the interconversion of carbon dioxide and water into bicarbonate and protons by carbonic anhydrase (CA). Ethoxzolamide (ETZ) inhibits CA and PhoPR regulon signalling [49]. Mtb undergoes reductive stress at acidic pH and relies on pathways that generate oxidized cofactors to mitigate this stress. Compounds that target thiol metabolism and redox homeostasis (AC2P20, AC2P36, 4-OH-OPB and auranofin) enhance reactive oxygen species (ROS) accumulation and exacerbate Mtb’s sensitivity to thiol-oxidative stress. Chloroquine (CQ) inhibits phagosomal acidification and disrupts pH- and redox-mediated drug tolerance [113]. Numerous compounds exhibit pH-dependent or enhanced activity at acidic pH and disrupt Mtb’s ability to maintain a neutral pH_IB_. These compounds (IDR-0020850, -0054790, -0099118, -0040669, -0081053, 1048, 20E11, 1G9, agrimophol) do not act as ionophores, suggesting that they target a protein important for maintaining pH_IB_. Only agrimophol has had its target (Rv3852) elucidated, but its function remains to be defined. Several compounds (nitazoxanide, compound 16 and monensin) lower pH_IB_ by interrupting Mtb’s ΔΨ and proton motive force (PMF). MarP is a serine protease that functions to maintain Mtb’s acid tolerance. BO43 directly targets MarP, also disrupting Mtb’s pH_IB_. Mtb undergoes metabolic remodelling at acidic pH. Isocitrate lyase (ICL) is induced in a pH-dependent manner and is inhibited by itaconic acid (ITA) and 3-nitropropionate (3 NP). ITA also disrupts pH_IB_, when given propionate as a carbon source. Dihydrolipoamide acyltransferase (DlaT) is inhibited by D157070 and is required for Mtb survival during infection [119], linking it to metabolic adaptation during environmental stress. C10 selectively reduces Mtb growth at acidic pH by inhibiting respirations and/or metabolism through an as yet unknown mechanism. Respiration and the ETC are probably modulated by acidic pH, and several compounds target ETC proteins including imidazopyradines (cytochrome *bc1-aa3*) and bedaquiline (BDQ) (ATP synthase). Some compounds (CLBQ14, compound 4, DPLG-2, and trifluoperazine) have their targets resolved and exhibit activity at acidic pH, but how they impact pH adaptation has yet to be defined. Together these compounds disrupt important pH-adaptation physiologies and serve to sensitize Mtb to acid stress.

Experimental findings show that the PhoPR regulon is strongly induced *in vitro* at pH 5.7, and induction of the regulon begins at the same pH (~6.4) where Mtb also begins to exhibit slowed growth [27]. The association of slowed growth with *phoPR* regulon induction and decreasing pH suggests that the PhoPR regulon plays a role in regulating pH adaptation (Fig. 1) [27]. Additionally, PhoPR regulates genes associated with carbon metabolism and redox homeostasis [25,38, 48], suggesting that *phoPR* plays a critical role in altering metabolic processes in response to acidic environments. Together, these findings link carbon source-dependent growth arrest with the induction of the PhoPR regulon and add another layer of regulation utilized by Mtb when exposed to an acidic environment.

**Table 1.** Compounds that target Mtb pH-driven adaptation

Compounds	Compound structure	pH-dependent activity*	Disrupts intrabacterial pH (pH_IB_)	Disrupts membrane potential	Mechanism of action	Reference(s)
AC2P20	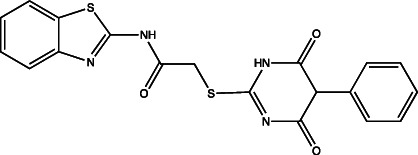	Selective	No	Undetermined	Covalent modification, formation of reactive oxygen species and depletion of free thiols	[69]
AC2P36	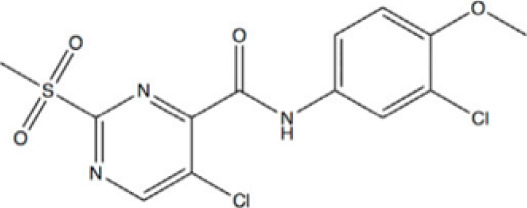	Enhanced	No	Undetermined	Covalent modification, formation of reactive oxygen species and depletion of free thiols	[70]
C10	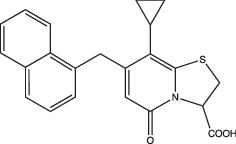	Selective	Undetermined	Undetermined	Inhibits respiration and/or metabolism	[96]
Bedaquiline	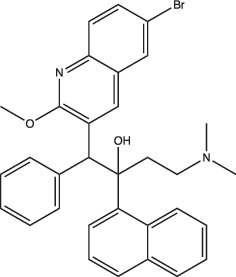	Non-specific	Yes	No	Inhibits the Mtb proton pump, ATP synthase	[86]
Auranofin	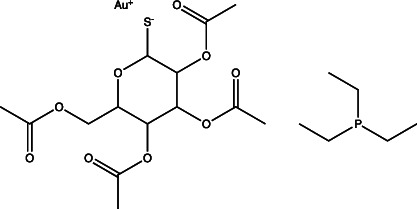	Non-specific	Undetermined	Undetermined	Inhibits the thioredoxin reductase enzyme (TrxB2), decreases free thiol concentrations	[98]
IDR-0054790	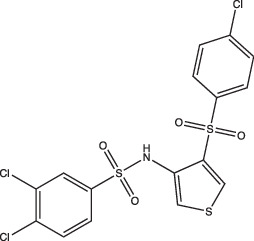	Selective	Yes	No	Undetermined	[72]
IDR-0099118	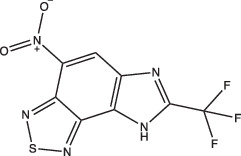	Selective	Yes	No	Undetermined	[72]
IDR-0040669	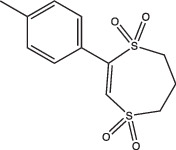	Enhanced	Yes	No	Undetermined	[72]
IDR-0081053	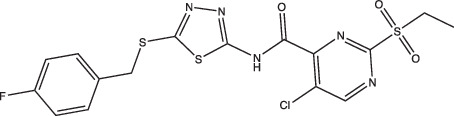	Enhanced	Yes	No	Undetermined	[72]
Ethoxzolamide	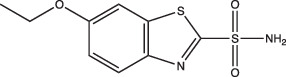	Non-specific	No	Undetermined	Inhibits PhoPR signalling, important TCS for pH-driven adaptations	[49]
Nitazoxanide	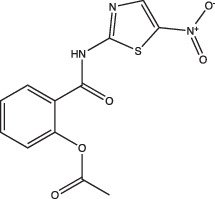	Enhanced	Yes	Yes	Stimulates autophagy and inhibits signalling by mTORC1, a major negative regulator of autophagy	[87,91]
Monensin	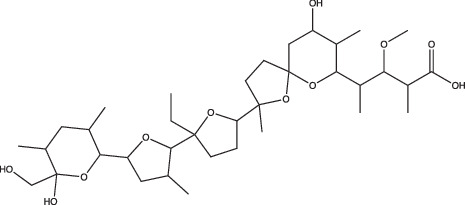	Undetermined, but is active at acidic pH	Yes	Yes	Sodium/hydrogen ionophore that disrupts pH_IB_ below limit of detection	[71]
1048	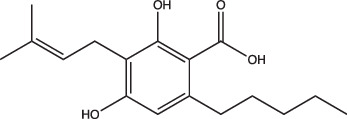	Selective	Yes	No	Undetermined	[71]
20E11	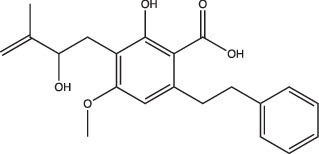	Selective	Yes	No	Undetermined	[71]
1 G9	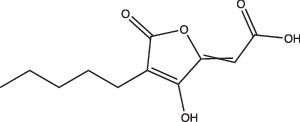	Selective	Yes	No	Undetermined	[71]
23 A6 (Agrimophol)	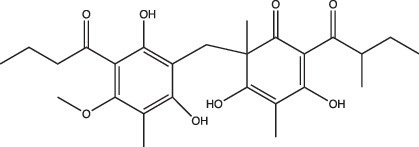	Enhanced	Yes	No	Targets Rv3852, protein of unknown function	[71,80]
4-OH-OPB	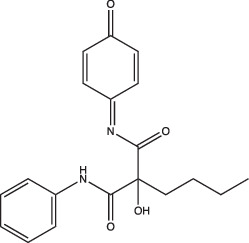	Selective	Undetermined	Undetermined	Covalent modification, formation of reactive oxygen species, and depletion of thiols and flavins	[99]
Trifluoperazine	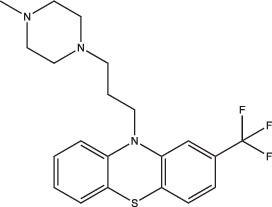	Enhanced	Undetermined	Undetermined	Inhibits protein and lipid synthesis, targets Rv1211, a calmodulin-like-protein that complexes with calcium	[100]
D157070	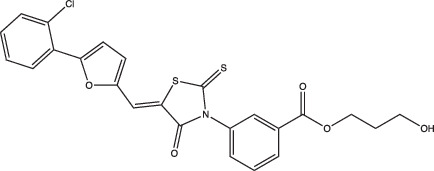	Non-specific, but requires non-replication at neutral and acidic pH	Undetermined	Undetermined	DlaT inhibitor, an enzyme that Mtb requires for resisting nitric oxide-derived reactive nitrogen intermediate stress	[101]
DPLG-2	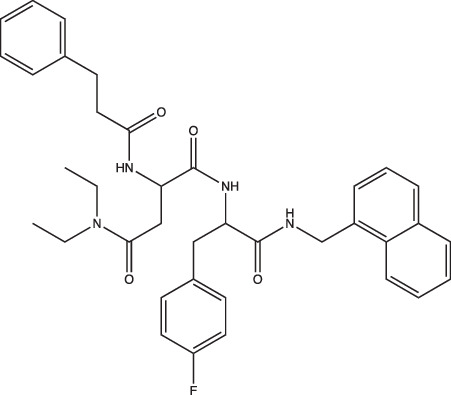	Undetermined, but is active at acidic pH with nitrosative stress	Undetermined	Undetermined	Mtb 20S proteasome inhibitor	[102]
8-Hydroxyquinoline	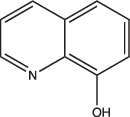	Non-specific	Undetermined	Undetermined	Undetermined	[103]
CLBQ14	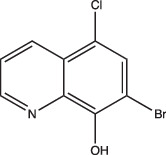	Undetermined, but is active at acidic pH	Undetermined	Undetermined	Targets Mtb’s methionine aminopeptidase	[104]
Compound 4	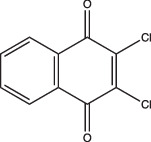	Undetermined, but is active at acidic pH	Undetermined	Undetermined	Targets Mtb’s methionine aminopeptidase	[105]
BO43	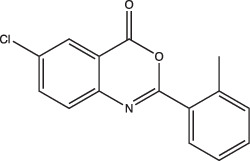	Selective	Yes	Undetermined	Inhibitor of MarP, acylates MarP, and lowers Mtb’s pH_IB_ and survival at low pH	[80]
Imidazopyradine series	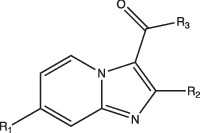	Undetermined, but is active at acidic pH	Yes	Undetermined	Targets QcrB, a component of the terminal cytochrome oxidase, and disrupts the electron transport chain	[88,89]
Itaconic acid	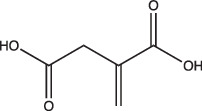	Selective	Yes, but only on propionate	Yes, but only on acetate or propionate	Itaconic acid covalently binds to isocitrate lyase, inhibiting its activity	[106,115]
3-Nitropropionate	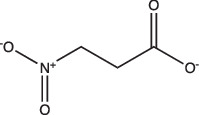	Selective	Undetermined	Undetermined	Inhibits succinate dehydrogenase activity (hypoxia) and isocitrate lyase activity (acidic pH)	[25,116, 117]
Compound 16	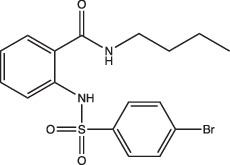	Selective	Undetermined	Yes	Undetermined	[93]
Chloroquine	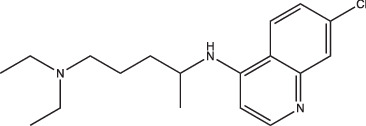	Undetermined	Undetermined	Undetermined	Inhibits phagosomal acidification, disrupts Mtb pH-and redox-dependent drug tolerance	[108,109, 113]

*pH-dependent activity categorized based on whether the compound exhibits (i) selective activity (only exhibits activity at acidic pH), (ii) enhanced activity (exhibits greater activity at acidic pH over neutral pH) or (iii) non-specific activity (active at both neutral and acidic pH) on Mtb growth under acidic conditions *in vitro.* Compound was listed as ‘active at acidic pH’ if acidic conditions were tested, even if pH-dependent activity of compound remains undetermined.

Transcriptional profiling is a valuable tool that can be utilized to identify whole system pathways and specific genes modulated by acidic pH. Several studies have used transcriptional profiling, microarray or RNA sequencing (RNAseq), to identify genes specifically regulated by acidic pH and/or conditional environments in concert with a pH-stress response [25,27, 36, 37, 49]. Fisher *et al.* were one of the first to analyse Mtb’s global transcriptional response to acidic pH using microarrays and real-time reverse transcription PCR and discovered 81 genes that were differentially expressed, including many involved in lipid metabolism [37]. Using microarrays as well, Walters *et al.* and Gonzalo-Asensio *et al.* both showed that PhoP positively regulated genes involved in lipid and carbon metabolism while Rohde *et al.* and Abramovitch *et al.* further revealed that the PhoPR regulon is induced during the initial stages of pathogenesis in macrophages, a mildly acidic environment [1,36, 38, 48]. RNAseq-based methods have helped elucidate pH-induced or repressed genes in a carbon-source-dependent or independent manner, as well as *phoPR*-dependent transcriptional changes in response to acidic pH [25,49]. Baker *et al.* showed that acid regulated genes are associated with carbon metabolism, lipid anabolism and replenishment of oxidized cofactors, supporting the previous connections made between acid-inducible and PhoPR-regulated genes [25]. In a recent study, the poorly understood two-component regulatory system, TcrXY, was shown to respond to pH [45]. Stupar *et al.* observed little overlap between the newly defined TcrXY 70-gene regulon and the previously established PhoPR regulon, noting that *tcrXY* appeared to regulate genes associated with long-term persistence and redox stress mitigation [45]. Together, transcriptional profiling can be used to identify key genetic regulators of pH-driven adaptation. In turn, these genetic elements can be developed into fluorescent transcriptional reporters for assessing gene expression in response to changes in the pH environment, like the CDC1551 (*aprA′*::GFP) reporter strain [27]. The *aprABC* locus is induced when exposed to low pH *in vitro* and in macrophages and is also dependent on PhoPR regulation, making it an ideal reporter candidate for examining pH and phagosomal-inducible transcriptional changes [27,50]. In addition, Stupar *et al.* generated a novel H37Rv (P*_tcrXY_*::mCherry; P*_rpoB_*::gfp) dual transcriptional reporter that also shows pH- and time-dependent [45] induction, supporting that transcriptional reporters are useful biomarkers to measure pH changes. Overall, transcriptional profiling is a useful tool for elucidating the metabolic requirements of Mtb undergoing acid stress, as well as understanding how pH-regulated genes are regulated in complex environments such as macrophage or animal infections.

## Genetic studies identifying mutants with altered pH-dependent adaptations

Establishing non-replicating persistence is important for Mtb to survive acid stress. However, a growing body of literature has revealed mutants that are capable of resisting acid stress or overcoming acid growth arrest altogether *in vitro*. These mutants can be leveraged to reveal mechanisms of physiological and genetic adaptation to acidic pH, and furthermore, could act as potential targets for TB drugs.

In early phases of macrophage infection, Mtb undergoes rapid replication which is ultimately deleterious to its survival and coincides with a decrease in overall bacterial viability [16]. It is not until Mtb enters a phase of slower cell division roughly 2 days following macrophage infection that the rate of killing begins to decrease. During this time of slowed growth, Mtb appears to adapt to the macrophage environment and establish a productive infection [16]. These observations are supported by computational modelling of the host immune response to Mtb infection where persistent infection and bacterial survival is contingent on establishing slow mycobacterial growth [51]. As previously mentioned, the mild acidity of the host macrophage is an important trigger for differential gene expression and Mtb intracellular survival. In *in vitro* stress models of Mtb growth at low pH in both rich and minimal media, Mtb will slow its growth or completely arrest growth altogether [24,27,35, 52]. Unlike other *in vitro* single stress models (i.e. starvation [53] and hypoxia [20]) where Mtb experiences physiological limitations that result in its cessation of growth, *in vitro* acid stress media and acid stress growth models contain all necessary nutrients and supplementation required to establish mycobacterial growth [32]. This observation suggests that pH-dependent cessation of growth may be genetically controlled.

We have pursued this hypothesis in our lab and shown that pH-dependent growth arrest is a suppressible phenotype, by conducting a genetic selection for mutants that can grow in minimal media buffered to pH 5.7, with glycerol as the sole carbon source [35,54]. This selection identified three independent amino acid substitutions (S211R, E215K and A228D) in PPE51 that allow for substantial growth to occur at acidic pH. These mutations were identified as dominant, gain-of-function mutations and regarded as enhanced acid growth (*eag*) mutants [35,54]. PPE51 is a mycobacteria-specific protein that is implicated in glycerol and nutrient uptake, an observation that has been studied by our lab as well as others [35,54,57]. PPE51 *eag* variants can grow specifically on glycerol, a carbon source that is normally non-permissive for growth at acidic pH, but the variants cannot grow on other non-permissive carbon sources such as glucose, lactate or propionate [25]. The *ppe51 eag* variants have enhanced glycerol uptake suggesting that growth arrest is caused by insufficient glycerol uptake to support growth in the selection conditions. Transcription of *ppe51* is induced at acidic pH, independent of growth arrest, in a *phoP*-dependent manner as well as 2 h post-infection in macrophages [25,36, 49]. Gouzy and colleagues observed that glycolytic carbon sources such as glycerol may limit Mtb growth at acidic pH through a mechanism of reduced glyceraldehyde 3-phosphate dehydrogenase (GAPDH) activity and accompanying reduction in glycolytic flux at acidic pH [34]. It is possible that *ppe51* is induced at acidic pH to attempt to compensate for reduced glycolytic flux, and that *ppe51 eag* variants overcome this barrier to growth by enhancing glycerol uptake.

Other mutants that allow for growth to occur *in vitro* in acidic media could also be described as *eag* variants. When *phoPR* is deleted, the mutant exhibits significantly enhanced growth on pyruvate as the sole carbon source at acidic pH when compared to WT Mtb [25]. Although pyruvate is permissive for WT Mtb growth at pH 5.7, the enhanced growth of *ΔphoPR* in the same culture conditions suggests that functional PhoPR is required to slow Mtb growth at acidic pH. Similarly, a *tgs1* mutant lacking the primary triacylglycerol synthase also exhibits enhanced growth in 7H9 medium adjusted to pH 5.5 [52]. While WT Mtb and the *tgs1* complement were able to replicate in the same culture conditions, the *Δtgs1* strain continued to grow more rapidly overall, providing another example of an *eag* phenotype. Baek and colleagues also showed that a *ΔdosR* mutant*,* the response regulator of the DosRST TCS and regulator of *tgs1* [58], shows a similar growth phenotype to *Δtgs1* under acid stress [52] and could also be described as having an *eag* phenotype as well.

Mutants have also been discovered that resist killing at acidic pH but cannot replicate. Tischler and colleagues showed that *ΔpstA2* and *ΔpstS1* exhibit enhanced resistance and cell viability in acidified 7H9 medium (pH 4.5) compared to the WT control [59]. While both *pstA2* and *pstS1* knockout mutants and the WT exhibited an overall decrease in bacterial viability at acidic pH, sensitivity to acidic pH was significantly more reduced in *ΔpstA2* and *ΔpstS1* compared to the WT. Both PstA2 and PstS1 are part of the Pst (phosphate-specific transport) uptake system in Mtb that transports inorganic phosphate (P_i_) [60]. More specifically, PstA2 is a membrane-spanning protein and PstS is a substrate-binding protein with high affinity for P_i_ [60]. It was proposed that WT Mtb may transport the monobasic form of phosphate and an additional proton, leading to acidification of the cytoplasm. In contrast, *ΔpstA2* and *ΔpstS1* Mtb might exhibit impaired protonated phosphate transport, resulting in fewer protons in the cytoplasm and increased acid resistance. Some other considerations for the growth of these mutants include the acidified medium which was buffered to pH 4.5. While Mtb is able to survive and maintain viability at pH 4.5 in phosphate-citrate buffered medium [24,61], the 7H9 medium used in this study contained albumin-dextrose-saline enrichment and Tween-80, which could potentially release free fatty acids that are toxic to Mtb at low pH [62,66]. However, Mtb can cease growth and maintain viability in 7H9 media containing oleic acid-albumin-dextrose-catalase enrichment and buffered slightly higher at pH 5.0 [27,34]. It is plausible that *ΔpstA2* and *ΔpstS1* Mtb may exhibit greater acid resistance and bacterial viability and growth in a different media type or a slightly less acidic media.

Transposon mutagenesis is a powerful approach that can be used to identify genes essential for survival during Mtb pH-dependent growth arrest and pH-driven adaptation. Transposon mutagenesis requires the construction of a transposon insertion library, which involves the relatively random integration of a transposon into a genetic element, thereby disrupting its function [67]. Vandal *et al.* used transposon mutagenesis to identify genes responsible for conferring acid resistance [24], by screening 10 100 Mtb transposon mutants in 96-well plates for their impaired ability to recover from exposure to 7H9 medium with Tween-80 buffered to pH 4.5. They identified 21 genes with independent transposon insertions that showed sensitivity to acidified 7H9 medium [24]. Two mutants (Rv2136c and MarP) maintained their sensitivity in 7H9 amended with Tyloxapol and phosphate-citrate buffer, both buffered to pH 4.5, and were also highly attenuated for virulence *in vivo* [23,24].

Chemical biology is a useful approach that can tackle the basic research aims of finding new pH-dependent physiologies, while also exploring the applied research potential of finding new therapeutics and novel mechanisms of action. Our lab’s discovery that ethoxzolamide (ETZ) inhibits the PhoPR regulon showed that chemical genetics can be used to identify physiologies important for Mtb survival at acidic pH. ETZ functions as a carbonic anhydrase (CA) inhibitor and revealed a potential link between carbon dioxide sensing, CA activity, PhoPR signalling and pH-dependent pathogenesis (Fig. 1) [49,68]. In another example of chemical biology approaches, compounds that are pH-selective Mtb growth inhibitors can be harnessed as chemical genetic tools for exploring pathways required for Mtb growth and survival at acidic pH. AC2P36 and AC2P20, both discovered by our lab, are pH-selective compounds that demonstrate Mtb sensitivity to thiol-oxidative stress at acidic pH (Fig. 1) [69,70]. Additionally, chemical probes can be powerful tools when coupled with previously mentioned genetic approaches, such as transcriptional profiling, to elucidate novel pH-responsive pathways. For example, the use of AC2P36 and AC2P20 in combination with transcriptional profiling at acidic pH is how we determined that both compounds were modulating redox and thiol homeostasis, sensitizing Mtb to chemical treatment (Table 1) [69,70]. Taken together, the independent approaches of transposon mutagenesis, transcriptional profiling and chemical biology can reinforce and complement each other to find new pH-driven adaptation pathways.

## Screening for compounds that inhibit Mtb pH-driven adaptation

Important goals of TB drug development include finding compounds that shorten the duration of treatment, improve safety and tolerability, provide greater efficacy, combat multidrug (MDR) and extensively drug-resistant (XDR) TB, and improve treatment options for latent TB infections. pH-driven adaptation is an attractive target for drug development efforts, and many TB researchers have developed methodologies or streamlined efforts for evaluating compounds that disrupt pathways required for Mtb to survive in acidic environments.

Two main screening methods are often used to identify anti-mycobacterial compounds: phenotypic screens against whole cells or *in vitro* target-based screens. Phenotypic whole-cell high-throughput screening (HTS) is an invaluable tool to rapidly identify hit compounds from chemical libraries. This approach has been adopted to identify compounds that specifically interfere with intrabacterial pH (pH_IB_) homeostasis [71,72]. Specifically, Darby and colleagues developed a whole-cell HTS method using Mtb expressing a pH-sensitive, ratiometric GFP (pHGFP) that allowed for measurements of pH_IB_ on live cells [71,73]. This study used whole-cell screening of a natural product library to identify disruptors of Mtb pH_IB_, and in doing so identified top four hit compounds: 1048, 20E11, 1G9 and 23A6 (agrimophol) (Fig. 1 and Table 1) [71]. Early *et al.* also capitalized on the use of pH-sensitive GFP and adapted it for a HTS of a diverse compound library against Mtb pH_IB_ which helped identify five top hit compounds: IDR-0020850, IDR0054790, IDR0099118, IDR-0040669 and IDR-0081053 (Fig. 1 and Table 1) [72,74]. While both studies successfully identified new disruptors of pH_IB_, pH-driven adaptation is not solely reliant on maintaining a hospitable pH_IB_. PhoPR plays a role in pH-driven adaptation, and directly induces ~50 pH-regulated genes, including the Acid and Phagosome Regulated locus, *aprABC* [21,27, 38, 48]. *aprABC*’s promoter is directly bound by PhoP and is induced in a pH-dependent manner and in macrophages [21,27, 75]. To identify chemical probes that inhibit the PhoPR regulon, our lab generated an acid-inducible biosensor strain by cloning the *aprA* promoter upstream of GFP, and used it to identify ETZ as an inhibitor of *phoPR* signalling (Fig. 1 and Table 1) [27,49]. RNAseq of ETZ-treated Mtb caused the downregulation (>2-fold, *P*<0.05) of 45 genes, all of which were also downregulated in the *phoP::*Tn mutant and confirmed that ETZ inhibits PhoPR regulon induction [49]. While ETZ is not growth inhibitory *in vitro*, it does reduce Mtb survival *in vivo*, showing that inhibition of pH-adaptation pathways required for virulence can be sensitized in multi-stress environments, further supporting that disrupting pH-adaptation pathways can be used for new drug development.

When a pH-dependent physiology or gene product is known, target-based screening can be a powerful tool for identifying active molecules. Maintaining intrabacterial pH homeostasis (pH_IB_) is critical for Mtb survival during acid stress. MarP is a transmembrane serine protease that is required for conferring acid resistance [24]. Catalytically inactive MarP fails to maintain pH homeostasis both *in vitro* and *in vivo*, sensitizing Mtb to acid stress [24,76]. MarP has been shown to cleave the peptidoglycan hydrolase RipA, an important enzyme for cell division [77]. In the same study, MarP-deficient Mtb cells exhibited cell elongation and impaired cell separation during acid stress [77]. Therefore, MarP is an attractive therapeutic target. Activity-based protein profiling (ABPP) utilizes small molecule probes, consisting of a target-specific reactive group and a reporter group, to identify potential protein binding partners [78]. For target-based HTS, an enzyme-specific probe tagged with a fluorophore (i.e. reporter) emits a strong signal when the reactive group covalently modifies the active site of its target protein [78]. However, in the presence of a competitor, the signal is decreased [79]. Using an ABPP approach, Zhao screened for inhibitors of MarP using the recombinant extracellular domain of MarP with binding affinity to a fluorophosphonate-rhodamine activity-based probe [78,80]. This allowed them to screen a >300 000 compound library and identify compounds that interfered with the binding of the probe to MarP’s serine hydroxyl, resulting in decreased probe fluorescence polarization. The screen discovered benzoxazinones as specific inhibitors of MarP, and further identified BO43 as a potent MarP inhibitor that disrupted Mtb’s pH_IB_ (Fig. 1 and Table 1) [80]. Other pathogenic mycobacterial species like *Mycobacterium avium* subsp. *paratuberculosis* also rely on a serine protease with over 92 % similar to Mtb’s MarP to maintain its pH_IB_, strongly suggesting that pH_IB_-disrupting chemicals like BO43 could eventually be co-opted to counteract acid resistance in multiple mycobacterial pathogenic species [81].

Additionally, this technology can be used to identify unknown targets of compounds identified from phenotypic HTS through an approach known as click chemistry ABPP (CC-ABPP). CC-ABPP involves the addition of alkyne or azide groups to a reactive group. This approach is useful for overcoming bulky groups that limit cell permeability and allows for probe labelling *in vivo* and reporter tagging *ex vivo* [78,79, 82]. As an example of its application, Zhao and colleagues used CC-ABPP by first generating a bioactive agrimophol alkyne, incubating it with *Mycobacterium bovis* BCG, and then applying a click-chemistry reaction with azido-biotin, allowing them to identify the BCG homologue of Mtb protein Rv3852 as the binding partner of the pH_IB_ inhibitor agrimophol (Fig. 1) [71,83]. Taken together, ABPP allows for screening and rapid observation of target-specific inhibitors and has already been shown to be a valuable approach for finding new inhibitors of pH-regulated genes required for Mtb’s survival.

## Classifying chemical probes by their pH-dependent activity or targets

A growing body of literature supports the classification of compounds that exhibit activity against Mtb and that are pH-dependent and/or target pH-dependent physiologies. These compounds can be further categorized by their general activity: (1) interruption of intrabacterial pH homeostasis (pH_IB_); (2) disruption of membrane potential; (3) activity as an ionophore; or (4) having other unique properties. Furthermore, not all compounds described herein strictly exhibit pH-dependent activity and some can still be active at both neutral and acidic pH. While their activity is non-specific across pH ranges, these compounds inhibit Mtb’s survival at acidic pH, with some targeting known pH-dependent physiologies. This demonstrates that the classification of compounds that disrupt Mtb’s survival at acidic pH remains broad and includes a diverse group of compounds.

Following the discoveries of genes required to maintain Mtb intrabacterial pH homeostasis, several studies sought to find inhibitors of pH_IB_ [71,72, 80]. Since the pH of the phagosome that Mtb resides in can range from mildly acidic (pH 6.2) to more strongly acidic (pH 4.5) [1,2, 84, 85], Mtb survival is dependent on its ability to sense external pH and maintain a relatively neutral internal pH to preserve its viability [24]. Thus, pH homeostasis is an attractive target because disrupting it at acidic pH can potentially sensitize Mtb to acid stress. MarP mutants provide compelling genetic evidence for this, as MarP mutants fail to maintain pH_IB_ in acid and are attenuated for virulence in *in vivo* [23,24]. In recent years, numerous compounds have been identified that disrupt Mtb pH_IB_: bedaquiline [86], IDR-0020850, IDR-0054790, IDR-0099118, IDR-0040669, IDR-0081053 [72], nitazoxanide [87], monensin [71], 1048, 20E11, 1G9, 23A6 [71], BO43 [80] and imidazopyradines [88,89]. Despite all these compounds disrupting pH_IB_, they are not structurally similar (Table 1). Furthermore, known mechanisms or targets of pH_IB_-disrupting compounds are also diverse. For example, bedaquiline and the imidazopyridine series both target major components of Mtb’s electron transport chain (Fig. 1); however, they target different components: the ATPase and QcrB, respectively. Additionally, not all pH_IB_ inhibitors are reliant on acidic pH conditions for activity. This is highlighted by bedaquiline, which does not exhibit pH-dependent activity; however, the IDR compounds rely on acidic pH conditions to exhibit either selective or enhanced activity (Table 1). Taken together, pH_IB_ inhibitor structure and diversity of activity suggests that there are many different pathways and genes regulating pH_IB_, and that distinct targets exist that can potentially sensitize Mtb to acid stress. Furthermore, these compounds can be useful tools to uncover new pathways and proteins important for maintaining pH_IB_.

The membrane potential (ΔΨ) and the transmembrane proton concentration gradient (ΔpH) are the two components that drive the proton motive force (PMF) (Fig. 1). It is important to make the distinction between compounds that disrupt membrane potential through a targeted mechanism or exhibit non-specific, depolarization of the membrane (i.e. ionophores). Compounds that affect the PMF via membrane potential disruption are attractive targets not only because it is essential for mycobacterial survival [24], but also because acidic pH has been shown to decrease Mtb’s membrane potential compared to neutral pH [90]. In addition to disrupting pH_IB_, nitazoxanide also reduces Mtb’s membrane potential, which is further augmented by acidic pH [87], and acts as a strong stimulator of autophagy and inhibitor of mTORC1 signalling [91]. Furthermore, its activity against replicating and non-replicating Mtb suggests that nitazoxanide has a potentially novel mechanism of action and multiple targets [87,92]. Compound 16 disrupts Mtb membrane potential in a pH-dependent manner, and has been proposed as a new tool to evaluate Mtb membrane potential disruption at acidic pH because it exhibits greater depolarization than CCCP compared to a DMSO control [93]. Monensin is another membrane potential disruptor that also acts as an ionophore (Table 1) [71]. While used as a general ionophore assay control, monensin does have therapeutic potential and has been used to treat *M. avium* subsp. *paratuberculosis* infections in cattle [94,95].

Another grouping of compounds are those that do not necessarily inhibit pH_IB_, disrupt membrane potential or act as ionophores as their proposed mechanism of action. Rather, they have unique or novel mechanisms of action and appear to disrupt functional pathways important for Mtb’s survival under acidic conditions. These compounds include AC2P20 [69], AC2P36 [70], C10 [96], auranofin [97,98], ethoxzolamide [49], 4-OH-OPB [99], trifluoroperazine [100], D157070 [101], DPLG-2 [102], 8-hydroxyquinoline [103], CLBQ14 [104], Compound 4 [105], itaconic acid [106], 3-nitropropionate [107] and chloroquine [108]. Specifically, there appears to be a subset amongst these compounds that are actively targeting molecules or pathways important for maintaining thiol and redox homeostasis in Mtb. AC2P20, AC2P36 and 4-OH-OPB (an oxyphenbutazone) all appear to have pH-dependent activity and covalently modify free thiols in Mtb, disrupting redox homeostasis and resulting in the formation of reactive oxygen species and depletion of these free thiol pools (Fig. 1 and Table 1) [69,70, 99]. This approach probably results in greater thiol-oxidative stress and further sensitizes Mtb to acidic pH. Auranofin, although it exhibits non-specific activity at both neutral and acidic pH, causes a decrease in free thiol concentrations by inhibiting TrxB2, a thioredoxin reductase (Fig. 1 and Table 1) [98]. Chloroquine (CQ) is an antimalarial agent that inhibits phagosomal acidification (Fig. 1 and Table 1) [109]. Its activity against Mtb has been attributed to multiple mechanisms: inhibiting macrophage efflux pumps, limiting iron availability and inhibiting phagosome–lysosome fusion [108,112]. Mishra and colleagues observed that acidification was required for redox-dependent multidrug tolerance, and that addition of CQ increased the killing efficacy of INH and RIF by five-fold [113]. These studies show compelling evidence that thiol-redox homeostasis has implications as a targetable pH-dependent physiology.

Other compounds in this grouping target unique physiologies completely. C10 inhibits respiration and metabolism through an undefined mechanism and decreases Mtb viability at acidic pH [96]. ETZ, a CA inhibitor, inhibits PhoPR signalling, an important TCS for regulating pH-driven adaptations (Fig. 1) [49]. ETZ inhibits Mtb CA activity in whole cells and Mtb survival in macrophages, but its exact mechanism of action in modulating Mtb physiology has yet to be fully elucidated (Fig. 1). Notably, carbon dioxide regulates PhoPR signalling independent of acidic pH, supporting a link between CA activity, carbon dioxide and PhoPR signalling [68]. Johnson *et al.* showed that ETZ does not reduce Mtb growth *in vitro* but does reduce Mtb growth in macrophages and mice. This is consistent with previous observations of *phoPR* knockout mutants, which are highly attenuated *in vivo* [39,114]. Likewise, itaconic acid is a covalent inhibitor of isocitrate lyase (ICL) activity in Mtb [106], and has been shown to disrupt Mtb pH homeostasis and membrane potential when grown on propionate or acetate (Fig. 1 and Table 1) [115]. 3-Nitropropionate (3 NP) is also a potent inhibitor of ICL activity [107]; however, data by Eoh and Rhee suggest that it may act preferentially on succinate dehydrogenase activity, rather than ICL activity [116]. 3 NP does inhibit recombinant Mtb ICL [117], and Baker *et al.* showed that 3 NP inhibits Mtb growth at acidic pH, but no change in growth at neutral pH, suggesting a pH-dependent requirement for ICL activity [25]. It is possible that 3 NP activity may be conditional and dependent on whether Mtb is undergoing hypoxia [116] or exposed to acidic pH [25]. ICL promotion of anaplerotic metabolism and strong induction by acidic pH makes itaconic acid and 3 NP useful tools to probe metabolic and pH-dependent pathways in Mtb. D157070 exhibits non-specific activity but can only do so under non-replicating conditions [101]. D157070 alone has no effect on Mtb viability at acidic pH, but synergistically kills Mtb under acidic conditions when nitrite is added because D157070 blocks resistance mechanisms needed to combat nitric oxide-induced stress [101]. Resistance to reactive nitrogen intermediates is mediated by an NADH-dependent peroxidase and peroxynitrite reductase system that is encoded by an alkyl hydroperoxide reductase subunit C (AhpC), an alkyl hydroperoxide reductase subunit D (AhpD), dihydrolipamide acyltransferase (DlaT) and lipoamide dehydrogenase (Lpd) [118,119]. D157070 directly targets DlaT, reducing Mtb viability under non-replicating conditions (Fig. 1) [101]. It should be noted that non-replicating conditions in this study utilized rich medium buffered to pH 5.5 [101], and that AhpCD, which complexes with DlaT, is induced at acidic pH [25], supporting that D157070 may act on pH-dependent metabolic pathways. DPLG-2, a proteasome inhibitor, is similar to D15070 in that it too exhibits activity at acidic pH in concert with nitrosative stress (Table 1) [102]. CLBQ14 and Compound 4 both target Mtb methionine aminopeptidases (Fig. 1) and are equally effective at inhibiting non-replicating Mtb in low pH, hypoxic medium compared to replicating Mtb [104,105]. Taken together, these compounds show that targets which are important for maintaining Mtb viability during acid stress are varied and distinct and that more consideration is needed for finding similar or novel physiologies altogether. Furthermore, there are still compounds which exhibit activity at acidic pH that have yet to be fully defined (i.e. trifluoperazine, 8-hydroxyquinoline) (Table 1).

Pyrazinamide (PZA) is a drug that has long been thought to be acidic pH-dependent, but its mechanisms of action and pH-dependence remain controversial. PZA is a prodrug whose activation to pyrazinoic acid (POA) is achieved through Mtb PncA, a nicotinamidase [120]. PZA exhibits strong *in vivo* activity and has long been regarded for decades as having activity at acidic pH but not neutral pH *in vitro* [121,122]. Previous reports suggested that PZA’s pH-dependent activity was due to the increased accumulation of POA, acting as an ionophore and uncoupler of the PMF, conferring cytoplasmic acidification [123,124]. In contrast, newer data suggest that PZA can sensitize Mtb at neutral pH when exposed to lower temperature, overexpression of PncA, nutrient-limited neutral pH medium or *in vivo* [9,125,128]. Peterson *et al.* also show that PZA/POA does not exhibit robust ionophore activity as previously thought, and that its anti-tubercular activity is independent of intrabacterial acidification [126]. Additionally, a new study by Fontes and colleagues may dispel previous reports of increased PZA activity at neutral pH, instead supporting that the acid–base equilibrium of POA drives the pH-dependence of PZA activity [124]. The study shows that when the pH of the medium is lowered, the equilibrium shifts from deprotonated, negatively charged POA towards protonated, neutral POA, which may act as an ionophore, uncoupling the PMF [124]. Fontes suggests that results by den Hertog *et al.* and Peterson *et al.* detailing PZA activity at neutral pH can be explained by the POA acid–base equilibrium, and proposes that the results of both studies are actually due to accumulation of protonated, neutral POA in solution and not anionic POA [124]. For this reason, the data surrounding PZA activity and its disputed impact on pH homeostasis are a developing and hotly debated area of study [124,129].

PZA resistance is also associated with coenzyme A (CoA) and fatty acid metabolism [128,130]. Thiede *et al.* found that genes required for cell envelope homeostasis and the response to cell envelope stress, including the alternative sigma factor SigE, are associated with PZA sensitivity [131]. Given that PhoPR is an important regulator of cell wall lipids (i.e. SL and acyltrehaloses) [48,132] which utilize CoA-containing precursors [31], and that it also functions in concert with SigE [43], it is possible that PhoPR-regulated, acid-responsive genes could have an impact on PZA activity. Indeed, mutants in the PhoPR-regulated putative nutrient transporter *ppe51* had increased susceptibility to PZA in infected mice [56]. Numerous mechanisms of action for PZA have been proposed, with a number of studies opposing said models [129]. Determining whether PZA has pH-dependent activity or acts as an ionophore, shows that classifying PZA and or other compounds in terms of how they target or modulate pH-dependent pathways is complex and open for interpretation. PZA remains part of the current therapy regimen to treat drug-sensitive, MDR and XDR TB [133]. This is in part due to PZA’s excellent lung tissue penetration among patients with a variety of different pulmonary TB lesion types and highlights its versatility in treating both drug-susceptible and drug-resistant TB in clinical settings [7].

## Combatting phenotypically drug-tolerant Mtb at acidic pH

Bacteria whose growth is halted by acidification of growth media, Mtb included, can become tolerant to antibiotics in a phenomenon known as phenotypic drug tolerance [134,137]. Previous work from our lab has shown that the *eag* variants in *ppe51* render Mtb susceptible to INH and RIF treatment specifically at acidic pH while the WT is able to persist under these treatment conditions [35]. Faster replication in macrophages is associated with enhanced killing [16]. In contrast, slower growth rates imposed by macrophage-derived pressures correlate with greater Mtb survival [51], supporting that *eag* variants have enhanced sensitivity to antibiotic treatment because they are unable to establish NRP. Likewise, PhoPR functions to slow Mtb growth at acidic pH, and knockout *phoPR* mutants are highly attenuated *in vivo* [39,114]. A recent study by Bellerose and colleagues showed that transposon mutants of *phoP* and *ppe51* were hypersensitive to multidrug treatment in mice [56]. The authors generated a *Δppe51* mutant and found that it was significantly more sensitive to PZA treatment during mouse infection compared to WT Mtb [56]. Together, these studies indicate a role for WT PPE51 and PhoPR in modulating Mtb adaptation to acidic pH and establishing phenotypic drug tolerance in Mtb.

Recent work by Mishra and colleagues show that acidic pH can also generate replicating, drug-tolerant Mtb [113]. They found that phagosomal acidification is required for establishing phenotypically drug-tolerant Mtb by altering its redox physiology, possibly mediated by PhoPR [25,113]. Interestingly, Mishra found that phagosomal acidification drives heterogeneity in the redox physiology of actively replicating Mtb, which exhibit a more reduced mycothiol redox potential and antioxidant capacity. Additionally, pharmacological disruption of phagosomal acidification with chloroquine (Fig. 1) was able to counteract drug tolerance *in vivo*, supporting a link between phagosomal pH, redox metabolism and phenotypic drug tolerance [113]. These data are consistent with findings by Liu *et al.*, who observed that enhanced drug tolerance in activated macrophages was driven in part by acidic pH [137].

Chemically disrupting pH-adaptation pathways to prevent Mtb from entering a state of non-replicating persistence or generating a reduced redox potential, and thus establishing drug tolerance, is a desirable achievement for future TB therapeutics [138]. Proof-of-concept for this approach was demonstrated for the drug chloroquine, which disrupts pH- and redox-homeostasis to kill Mtb [113]. Phenotypic whole-cell HTS and target-based screening methods can be readily adapted in future studies to find compounds that inhibit Mtb phenotypic drug tolerance at acidic pH. Similarly, these approaches can also be harnessed to find new compounds that target acid adaptation pathways which may render Mtb hypersensitive, specifically in combination with existing anti-TB drugs like PZA. Given that ETZ inhibits *phoPR* regulon induction, it would be interesting to see whether combinatorial therapy of ETZ and PZA could yield similar hypersensitivity that was observed in Mtb mutants lacking functional *phoP* [56]. Shortening TB therapy is a key challenge in combatting the TB epidemic, and it is possible that targeting pH-dependent physiologies will play an important role in defining new, shorter treatment regimens.

## Abbreviations

ABPP: activity-based protein profiling
CA: carbonic anhydrase
CA: PMF proton motive force
CC: click chemistry
CQ: chloroquine
ETZ: ethoxzolamide
HTS: high-throughput screening
ICL: isocitrate lyase
MDR: multidrug resistant
Mtb: *Mycobacterium tuberculosis*
3-NP: 3-nitropropionate
NRP: non-replicating persistent
OA: oxaloacetate
PEP: phosphoenolpyruvate
pHIB: intrabacterial pH
POA: pyrazinoic acid
RNAseq: RNA sequencing
TAG: triacylglycerol
TB: tuberculosis
WT: wild-type
XDR: extensively drug resistant
